# Supplementary material for: Measuring hierarchical structure across hominid percussive tool-use sequences
Source: Commun Biol. 2026 Feb 19;9:457. doi: 10.1038/s42003-026-09633-8 (PMC13031648; doi:10.1038/s42003-026-09633-8)
Supplement: Supplementary file 2 — Description of Additional Supplementary Files [file 42003_2026_9633_MOESM2_ESM.pdf]

Description of Additional Supplementary Files:

File name: Supplementary Data 1

Description: Data for all analyses and figure creation.

File name: Supplementary Code 1

Description: Jupyter Notebook file containing instructions and code for running full measurement pipeline.

File name: Supplementary Code 2

Description: R code for running the analyses reported in the main manuscripts

File name: Supplementary Code 3

Description: R code for creating the figures included in the main manuscript.
